# Supplementary material for: Reconciling Mining with the Conservation of Cave Biodiversity: A Quantitative Baseline to Help Establish Conservation Priorities
Source: PLoS One. 2016 Dec 20;11(12):e0168348. doi: 10.1371/journal.pone.0168348 (PMC5173368; doi:10.1371/journal.pone.0168348)
Supplement: S1 Dataset — (ZIP) [file pone.0168348.s002.zip › Taxa/Serra Norte/SN_2013/N4E/Lista N4E-19.pdf]

CAVIDADE N4E-0019

Classe	Ordem	Fam/Outros	Gên/Outros	Espécie	S-U	U-S	Zon.
Annelida	Oligochaeta			jovem		1	P
Arachnida	Acari	(Parasitengonina)		sp.9		X	P
Arachnida	Acari	Argasidae	*Ornithodoros*	sp.1	X	X	A
Arachnida	Acari	Prostigmata		sp.	X		
Arachnida	Acari	Trombiculidae		sp.39	X	X	P/A
Arachnida	Amblypygi	Phrynidae	*Heterophrynus*	*longicornis*	X		
Arachnida	Araneae	Ctenidae		jovem		1	E
Arachnida	Araneae	Dipluridae		jovem		1	E
Arachnida	Araneae	Filistatidae		jovem	X	X	E
Arachnida	Araneae	Ochyroceratidae	*Ochyrocera*	sp.1	X	X	P/A
Arachnida	Araneae	Oonopidae		jovem		X	P
Arachnida	Araneae	Pholcidae	*Mesabolivar*	sp.1	X	X	E
Arachnida	Araneae	Pholcidae	Ninetinae	sp.1		X	E
Arachnida	Araneae	Salticidae		jovem	X	X	E
Arachnida	Araneae	Scytodidae	*Scytodes*	sp.	X		
Arachnida	Araneae	Theridiidae	*Theridion*	sp.	X		
Arachnida	Araneae	Theridiosomatidae	*Plato*	sp.1	X	X	P
Arachnida	Opiliones	Escadabiidae		sp.2		X	P/A
Arachnida	Pseudoscorpiones	Chernetidae	*Spelaeochernes*	sp.1	X	X	P
Arachnida	Pseudoscorpiones	Chthoniidae		jovem		X	A
Chilopoda	Scutigeromorpha	Pselliodidae	Sphendononema	*guildingii*	X	2	A
Diplopoda	Polydesmida	Chelodesmidae		jovem		2	A
Diplopoda	Polydesmida	Fuhrmannodesmidae		jovem		X	P
Diplopoda	Polydesmida	Pyrgodesmidae		sp.1	X	X	A
Diplopoda	Spirostreptida	Pseudonannolenidae	*Pseudonannolene*	jovem		4	P/A
Entognatha	Collembola	Isotomidae		sp.1		X	P
Entognatha	Collembola	Paronellidae		sp.1		X	P
Entognatha	Collembola	Sminthuroidea		sp.2	X	X	P
Entognatha	Diplura	Campodeidae		sp.1	X	X	E
Entognatha	Diplura	Projapygidae		sp.1		X	E
Insecta	Blattodea			jovem	X	2	E/P
Insecta	Coleoptera	Bostrichidae		sp.1	X		
Insecta	Coleoptera	Hydrophilidae		sp.9		X	E
Insecta	Coleoptera	Nitidulidae		sp.	X		
Insecta	Coleoptera	Staphylinidae		sp.39	X	X	P
Insecta	Diptera	Aulacigastridae		sp.		X	E
Insecta	Diptera	Mycetophilidae		sp.	X		
Insecta	Diptera	Psychodidae	*Deanemyia*	*samueli*	X		
Insecta	Diptera	Psychodidae	Phlebotominae	sp.		X	P/A
Insecta	Diptera	Psychodidae	*Sciopemyia*	*sordellii*	X		
Insecta	Diptera	Psychodidae	*Telmatoscopus*	sp.		X	P
Insecta	Diptera	Tipulidae	Tipulinae	sp.	X	X	E
Insecta	Heteroptera	Cydnidae		jovem		X	P
Insecta	Heteroptera	Reduviidae	Emesinae	sp.	X		
Insecta	Heteroptera	Reduviidae	*Zelurus*	sp.	X	2	E
Insecta	Hymenoptera	Formicidae	*Camponotus*	sp.2		X	E
Insecta	Hymenoptera	Formicidae	*Crematogaster*	sp.	X		
Insecta	Hymenoptera	Formicidae	*Dolichoderus*	aff. *bispinosus*	X		
Insecta	Hymenoptera	Formicidae	*Gnamptogenys*	sp.1		X	P
Insecta	Hymenoptera	Formicidae	*Gnamptogenys*	*striatula*	X		
Insecta	Hymenoptera	Formicidae	*Paratrechina*	sp.	X		
Insecta	Isoptera	Termitidae		sp.	X	X	
Insecta	Lepidoptera	Tineoidea		sp.4	X	X	E
Insecta	Orthoptera	Phalangopsidae	*Paraclodes*	sp.	X		
Insecta	Orthoptera	Phalangopsidae	*Phalangopsis*	sp.	X		

Insecta	Psocoptera	Psocomorpha		jovem	X		
Insecta	Thysanura	Nicoletiidae	Nicoletiinae	sp.1	X		
Gastropoda	Pulmonata	Bulimulidae	*Naesiotus*	sp.		X	P
Gastropoda	Pulmonata	Subulinidae	*Leptinaria*	sp.	X		
Malacostraca	Isopoda	Scleropactidae	*Circoniscus*	*buckupi*	X		
Symphyla		Scutigerellidae	*Hanseniella*	sp.	X	X	P
Amphibia	Anura			sp.	1		A
Mammalia	Chiroptera	Emballonuridae	*Peropteryx*	sp.	5		A
